# Risk factors for infection after carbapenem-resistant *Acinetobacter baumannii* colonization

**DOI:** 10.1007/s10096-024-04936-2

**Published:** 2024-09-16

**Authors:** Maddalena Peghin, Filippo Givone, Maria de Martino, Raja Waqar Ali, Elena Graziano, Miriam Isola, Paolo Antonio Grossi

**Affiliations:** 1 Infectious and Tropical Diseases Unit, Department of Medicine and Surgery, University of Insubria-ASST-Sette Laghi, Varese, Italy; 2 Division of Medical Statistic, Department of Medicine (DAME), University of Udine, 33100 Udine, Italy

**Keywords:** *Acinetobacter baumannii*, Colonization, Multidrug-Resistant, Carbapenem-resistant *Acinetobacter baumannii*

## Abstract

**Purpose:**

Predicting infection risk in carbapenem-resistant Acinetobacter baumannii (CRAB) colonized patients may help in improving timely appropriate antibiotic therapy. This study aims to explore risk factors for developing infections in hospitalized patients with previous CRAB colonization.

**Methods:**

We performed an observational retrospective cohort study at ASST Sette Laghi-Varese Hospital between January 2020 and December 2022. All consecutive adult (> 18 years old) hospitalized patients with documented colonization by CRAB at any anatomical site or with CRAB infections preceded by CRAB colonization were included. Univariate and multivariate analyses were performed to investigate infection risk factors.

**Results:**

Overall, 144 patients were included in the study: 104 colonized only and 40 infected patients. Colonization and infection rates significantly changed over the years (2020–2022, p < 0.001). The incidence of infections in CRAB carriers was 27.8% (40/144). Median time from colonization to infection was 4 days (IQR 1-8.5). Overall, inhospital mortality was 32.7% and 55.0% in colonized only and infected patients, respectively. At the multivariable logistic regression cardiovascular disease (OR 5.83, 95% CI 1.12–30.43, p = 0.037), COVID-19 (OR 3.72, 95% CI 1.16–11.91, p = 0.027) and intensive care unit (ICU) admission (OR 8.83, 95% CI 2.94–26.51, p < 0.001) were risk factors independently associated with cardiovascular disease CRAB infection after colonization.

**Conclusions:**

We observed an increased infection risk in patients colonized with CRAB with cardiovascular disease, COVID-19 and admitted in ICU setting. Additional evidence is needed to identify predictors of infection in colonized patients.

**Supplementary Information:**

The online version contains supplementary material available at 10.1007/s10096-024-04936-2.

## Introduction

Antimicrobial resistance (AMR) is an emerging threat to public health care systems worldwide [1]. The coronavirus disease 2019 (COVID-19) pandemic triggered and expanded AMR diffusion due to the pressure on healthcare systems, increased rates of irrational use of antimicrobials, and discontinuation of infection control programs [2, 3].

Multidrug-resistant (MDR) bacteria carriage might favor the spread of AMR and might increase the risk of subsequent infection by the colonizing pathogen over time, but the magnitude of this risk is poorly understood [4]. The available knowledge of the incidence of infection in MDR colonized patients is still limited and has been mostly focused on specific populations of immunocompromised hosts and critically ill patients and specific pathogens, including mostly extended-spectrum β-lactamase-producing *Enterobacterales* (ESBL), carbapenem-resistant *Enterobacterales* (CRE) and vancomycin-resistant *Enterococcus* (VRE) [5].

Carbapenem-resistant *Acinetobacter baumannii* (CRAB) has gained global priority for research and development of new antibiotics as a critical nosocomial pathogen, causing hospital outbreaks and infections with high mortality rates [1, 6–9]. Most information about CRAB infections is based on data from outbreak investigations and complex patients in the intensive care unit (ICU) setting [10–12]. A better knowledge of CRAB colonization dynamics and a more accurate prediction of which CRAB carriers are at risk of CRAB infections, both in ICU and general ward settings, could help in improving preventive measures or timely appropriate antibiotic therapy.

The aim of this study was to explore risk factors for developing infections in all hospitalized patients with previous CRAB colonization.

## Materials and methods

### Setting, patients and data collection

**Fig. 1:**
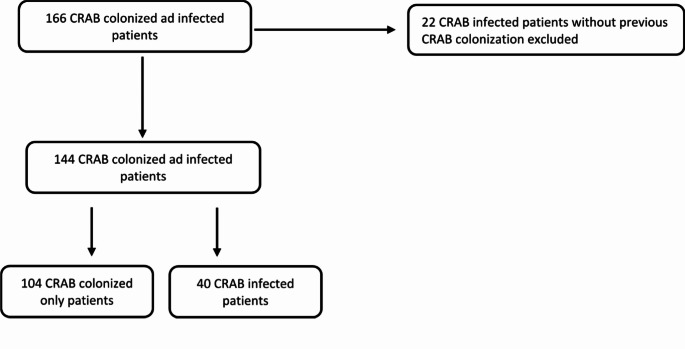
Flow diagram of CRAB colonized ad infected patients included in the study (2020–2022)

We performed an observational retrospective cohort study at ASST Sette Laghi – Varese Hospital between January 2020 and December 2022 according to the Strengthening the Reporting of Observational Studies in Epidemiology Statement (Supplementary Table 1). All consecutive adult (> 18 years old) hospitalized patients with documented colonization by CRAB at any anatomical site or infections preceded by CRAB colonization, and detected after hospital admission, during the study period, were eligible. Patients were included only once at the time of the first CRAB detection. Patients with CRAB infection without previous CRAB colonization were excluded from the study (Fig. 1).

A database concerning demographic, clinical, laboratory and antimicrobial data was drawn up. Computerized hospital databases or clinical charts were reviewed by trained doctors using a pre-established questionnaire. Information was anonymously recorded in an electronic database and included demographics, comorbidities, recent hospitalization (in the previous 3 months), recent antibiotic therapy (in the previous 3 months), surgery (in the previous 3 months), SARS-CoV-2 infection, cause of hospitalization and ICU admission, laboratory and clinical data on the day of colonization and of CRAB infection onset, site of colonization, timing of colonization and CRAB infection onset, time from colonization to CRAB infection onset, microbiological data and antibiotic regimens in the CRAB infection group, duration of ICU and hospital stay, clinical cure, all causes 30 day mortality 30 from colonization and CRAB infection onset. Risk variables were collected on the colonization or infection day.

### Endpoints of the study

The primary endpoint of the study was to describe the incidence of CRAB infection in hospitalized patients with CRAB colonization and to explore risk factors for developing infections CRAB colonized patients. The secondary endpoint was to describe patients’ comorbidities, cause of hospital admission and clinical outcome of CRAB infected and colonized patients in the whole hospital setting.

### Study definitions and screening

Based on hospital routine practice, all patients admitted to the intensive care unit (ICU) were subjected to collecting nasal swabs, rectal swabs, axillary swabs, inguinal swabs, respiratory sample cultures, and urine cultures within 48 h of admission and thereafter weekly. For patients hospitalized in non-ICU wards, microbiological screening was performed according to clinical judgment or epidemiological reasons.

CRAB colonization was defined as the growth of CRAB from clinical specimens from any anatomical site in the absence of clinical manifestations of infection. Multisite colonization was defined as a positive culture from more than one specimen from different anatomical sites in the same patient during hospitalization before infection onset. CRAB infection was defined as the growth of CRAB from clinical specimens in the presence of clinical signs and symptoms consistent with infection. Infections were diagnosed and classified according to Centers for Disease Control and Prevention *(*CDC*)/* National Healthcare Safety Network (NHSN) in hospital-acquired pneumonia (HAP), ventilator-associated pneumonia (VAP), bloodstream infections (BSI) and others (urinary tract infections, skin and soft tissue infections, intra-abdominal infections) [13]. Subsequent CRAB infection referred to CRAB infection developed after colonization.

Bacteria were classified as MDR, extensively drug-resistant (XDR) and pandrug-resistant (PDR) according to the susceptibility test [14]. The burden of comorbidities was estimated with Charlson comorbidity index (CCI) [15]. Shock was defined according to the international consensus guidelines [16].

Patients completed follow-up until discharge or until death. All-cause mortality at 30 days after documented colonization and in-hospital mortality were recorded. Persistence of any positive culture > 7 days after the beginning of targeted therapy was defined as microbiological failure.

### Microbiological identification and testing

The identification of CRAB strains was based on local microbiology laboratory standards. Isolated colonies from blood cultures or other positive cultures were identified by Matrix Assisted Laser Desorption Ionization Time of Flight Mass Spectrometry MALDI-TOF MS system and antimicrobial susceptibility was tested using the VITEK 2 system (bioMérieux, Inc., Marcy l’Étoile, France) according to the manufacturer’s instructions. The determination of cefiderocol and ampicillin/sulbactam susceptibility tests were not available. The MICs of antibiotics were classified according to the breakpoints established following the European Committee on Antimicrobial Susceptibility Testing (EUCAST) criteria [17].

### Antibiotic treatment

The empirical antibiotic therapy was defined as the initial antibiotic regimen started within 24 h of infection suspicion. *A*ppropriate empirical therapy was defined as the use of at least one in vitro active drug within the first 24 h from infection onset. The target antibiotic regimen was defined as an antibiotic regimen chosen based on the definitive susceptibility test and according to clinical judgment by an infectious disease consultant or the attending physician. Definitive target treatment was included in the analysis if it was administered for at least 24 h. Treatment regimens were classified as either monotherapy or combination therapy, depending on the number of drugs administered.

The usual antimicrobial dosages, (with adjustments for renal impairment made according to manufacturer recommendations) were the following: ampicillin/sulbactam, dosage of 3 g every 6 h; colistin, loading dose of 9 million IU followed by 4.5 million IU every 12 h; tigecycline, loading dose of 200 mg followed by 100 mg every 12 h; gentamicin, dosage of 5 mg/kg every 24 h; rifampin, dosage of 10 mg/kg/day; trimethoprim/sulfamethoxazole, dosage of 15–20 mg/kg/day divided into doses every 6 h; Fosfomycin, dosage of 16 g/day divided every 6–8 h; meropenem, dosage of 1–2 g every 8 h; cefiderocol, dosage of 2 g every 8 h.

### Ethics

The study was conducted according to the principles stated in the Declaration of Helsinki. The local ethics committee approved the study protocol.

### Statistical analysis

Absolute values, percentages, mean and median (standard deviation (SD) or interquartile range (IQR)) were calculated. Categorical variables were compared using the chi-squared test or Fisher’s exact test, while continuous variables were compared using a Student t-test or Mann-Whitney U test, according to the Shapiro-Wilk test establishing whether data were normally or non-normally distributed. Cochran-Armitage test was applied to assess any trend in terms of colonization and infection rates across the years. A univariable and multivariable logistic regression was performed to establish risk factors associated with the progression from CRAB Colonization to CRAB Infection, estimating the odds ratios (OR; 95% CI). The variables included in the multivariable analysis were chosen from the statistically significant variables in univariable analysis (*p* < 0.05) and they were selected for their clinical significance, considering the number of events and potential collinearities (for example ICU, CVC, and mechanical ventilation). The performance of the model was assessed using the area under the ROC curve and the calibration belt test [18]. No imputation of missing data was performed. Analyses were performed by STATA 18.

## Results

### Setting and study population

Overall, during the study period, 144 patients were included in the study: 104 (72.2%) CRAB colonized-only and 40 (27.8%) CRAB infected patients (Fig. 1). A sample size of 144 patients produces a two-sided 95% confidence interval (CI) with a precision of 7.5% for the proportion of CRAB infected patients.

Colonization and infection rates significantly changed over the years (2020: 10 hospital admissions per 1,000 person-years, 2021: 71 hospital admissions per 1,000 person-years; 2022: 27 hospital admissions per 1,000 person-years; *p* < 0.001). The overall incidence density of CRAB colonization and infection based on ICU-admissions was 0.7%.

Baseline demographic and clinical characteristics are summarised in Table 1. In brief, the median age was 71 years (IQR 60.5–78) and 43 (29.9%) were female. The median CCI was 4 (IQR 2–5). A total of 92.4% (133) reported at least one baseline co-morbidity, most often cardiovascular disease (116, 80.6%) and neurological disorders (34, 23.6%). Overall, 36.1% (52) of the cohort was positive for SARS-CoV-2. Around 86 (59.7%) patients had a history of recent antibiotic treatment and 84 (58.3%) patients had a recent hospital admission in the previous 3 months (Table 1). Around 94 (65.3%) were initially admitted in a general ward and 50 (34.7%) in ICU setting. Overall, in-hospital mortality was 32.7% and 55.0% in colonized only and infected patients, respectively (Table 1). Comparison between patients admitted to ICU and non-ICU wards is described in Supplementary Table 2.

**Table 1:** General characteristics of study population and outcome: comparison between colonized-only and infected patients (*n* = 144)

Characteristics	General population *N* = 144	Colonized-only patients *N* = 104	Infected patients *N* = 40	*P* value
Age, years, median (IQR)	71 (60.5–78)	73 (61–80)	67 (60-72.5)	0.007
Gender, female	43 (29.9%)	33 (31.7%)	10 (25.0%)	0.43
CCI median (IQR)	4 (2–5)	4 (2–6)	3 (2–4)	0.005
Number of comorbidities, mean±SD	2.0±1.2	1.9±1.2	2.3±1.1	0.15
Comorbidities				
Cardiovascular disease	116 (80.6%)	79 (76.0%)	37 (92.5%)	0.025
Neurological disorders	34 (23.6%)	33 (27.0%)	6 (15.0%)	0.12
Diabetes	34 (23.6%)	19 (18.3%)	15 (37.5%)	0.015
COPD	11 (7.6%)	8 (7.7%)	3 (7.5%)	0.97
Cerebrovascular disease	27 (18.8%)	23 (22.1%)	4 (10.0%)	0.095
Obesity (BMI ≥ 30 kg/m2)	17 (11.8%)	4 (3.8%)	13 (32.5%)	< 0.001
Chronic kidney disease	28 (19.4%)	22 (21.2%)	6 (15.0%)	0.40
Chronic liver disease	5 (3.5%)	3 (2.9%)	2 (5.0%)	0.53
Solid organ transplant	2 (1.4%)	1 (1.0%)	1 (2.5%)	0.48
HIV	1 (0.7%)	1 (1.0%)	0 (0.0%)	0.53
Previous hospitalization	84 (58.3%)	63 (60.6%)	21 (52.5%)	0.38
Previous antibiotic treatment	86 (59.7%)	62 (59.6%)	24 (60.0%)	0.97
Previous surgery	46 (31.9%)	39 (37.5%)	7 (17.5%)	0.021
Department of hospitalization				< 0.001
Non-ICU ward	94 (65.3%)	104 (85.2%)	8 (20.0%)	
ICU ward	50 (34.7%)	18 (17.3%)	32 (80.0%)	
Cause of hospital admission				< 0.001
COVID- 19	52 (36.1%)	22 (21.2%)	30 (75.0%)	
Infection	16 (11.1%)	15 (14.4%)	1 (2.5%)	
Trauma	8 (5.6%)	7 (6.7%)	1 (2.5%)	
Cardiovascular disease	12 (8.3%)	11 (10.6%)	1 (2.5%)	
Neurological disease	9 (6.2%)	7 (6.7%)	2 (5.0%)	
Gastrointestinal disease	15 (10.4%)	16 (13.1%)	1 (2.5%)	
Lung disease	13 (9.0%)	10 (10.7%)	3 (7.5%)	
Oncohematological disease	4 (2.8%)	4 (3.9%)	0 (0%)	
Other	10 (6.9%)	12 (9.6%)	0 (0%)	
Mechanical ventilation	47 (32.6%)	17 (16.3%)	30 (75.0%)	< 0.001
CVC	52 (36.1%)	17 (16.3%)	35 (87.5%)	< 0.001
Sites of colonization, median (IQR)	2 (1–4)	2 (1–3)	3 (2-5.5)	0.003
Multisite colonization	87 (60.4%)	56 (53.8%)	31 (77.5%)	0.009
Time from admission to colonization	9 (4–24)	9 (2–26)	9 (6-20.5)	0.71
Time from colonization to infection, median (IQR)			4 (1-8.5)	
Hospitalization length (days), median (IQR)	32 (18-52.5)	31 (16.5–49.5)	36.5 (27–57)	0.049
30-day mortality from colonization	55 (38.2%)	36 (34.6%)	19 (47.5%)	0.15
In-hospital mortality	56 (38.9%)	34 (32.7%)	22 (55%)	0.014

*Abbreviations* BMI, body mass index; CI, confidence interval; CCI, Charlson Comorbidity Index; COPD, Chronic Obstructive Pulmonary Disease; COVID-19. Coronavirus disease; CVC, Central Venous Catheter; HIV, Human Immunodeficiency Virus; ICU, Intensive Care Unit, IQR, interquartile Range

### Dynamics of CRAB colonization and infection

CRAB carriage was detected in 6.9% (10/144) of patients on hospital admission, whereas 93.1% (134/144) of patients acquired CRAB carriage during the hospital stay. Around 60.4% (87/144) of patients presented multisite CRAB colonization, with a median of 2 colonized sites. Median time from hospital admission to CRAB colonization was 9 days (IQR 4–24). The incidence of infections in CRAB carriers was 27.8% (40/144). Median time from colonization to infection was 4 days (IQR 1-8.5). Most patients (32/40, 80%) developed infection in the ICU setting.

Median time from hospital admission to CRAB infection was 17 days (IQR 9–27). Overall, 60% (24/40) of patients had a VAP, 32.5% (13/40) had a BSI, 5% (2/40) had a HAP and 2.5% (1/40) had a surgical site infection due to CRAB (Supplementary Table 3). Four (2.8%) patients developed septic shock.

As for microbiological data, 94% of the *A. baumannii* strains isolated showed an XDR resistance profile. Empirical treatment was appropriate only in 2 patients. Overall, 38 out of 40 infected patients received definitive target treatment, because 2 patients died before receiving any active antibiotic therapy. Median time from the onset of infection to appropriate treatment was 2 days (IQR 0–3). All patients received a combination regimen, mainly with three drugs (27/40, 67.5%). Overall, most patients were treated with a backbone regimen of ampicillin/sulbactam and colistin in association with a third drug (meropenem or rifampin) (Supplemental Table 4). Four patients received cefiderocol after failure of first-line treatment and 2 as first-line therapy. The median duration of targeted therapy was 11 days (range 3–15) days.

As regards the development of adverse pharmacological effects, renal impairment was observed in 5 out of 40 patients (12.5%) and all patients were under a colistin-based regimen. No hepatic or neurological toxicities were recorded. Microbiological eradication was observed in 60% (15/25) of patients with available cultures.

### Risk factors for CRAB infection onset after CRAB colonization

In the univariate analysis, individual risk factors for transitioning from colonization to infection in patients with CRAB were cardiovascular disease (OR 3.90, 95% CI 1.11–13.75, *p* = 0.034), diabetes (OR 2.68, 95% CI 1.19–6.04, *p* = 0.017), obesity (OR 12.03, 95% CI 3.63–39.91, *p* < 0.001), COVID-19 (OR 11.18, 95% CI 4.75–26.33, *p* < 0.001), number of colonization sites (OR 1.14, 95% CI 1.02–1.28, *p* = 0.022), use of central venous catheter (CVC) (OR 35.82, 95% CI 12.27–104.60, *p* < 0.001), mechanical ventilation (OR 15.35, 95% CI 6.34–37.18, *p* < 0.001) and ICU ward (OR 19.11, 95% CI 7.57–48.27, *p* < 0.001). Age (OR 0.97, 95% CI 0.94–0.99, *p* = 0.019), CCI (OR 0.76, 95% CI 0.63–0.92, *p* = 0.006), and previous surgery (OR 0.35, 95% CI 0.14–0.87, *p* = 0.025) resulted as a protective factor from CRAB colonization to infection at univariate analysis (Table 2).

**Table 2:** Univariable and multivariable analysis of risk factors associated with progression from CRAB colonization to CRAB infection (*n* = 144)

Risk factors	OR	Univariate 95% CI	*p*-value	OR	Multivariate 95% CI	*p*-value
Gender- Female	0.72	0.31,1.64	0.430			
Age	0.97	0.94,0.99	0.019	0.96	0.91,1.02	0.188
CCI	0.76	0.63,0.92	0.006	0.88	0.59,1.32	0.543
Number of comorbidities	1.24	0.92,1.68	0.155			
Cardiovascular disease	3.90	1.11,13.75	0.034	5.83	1.12,30.43	0.037
Diabetes	2.68	1.19,6.04	0.017	2.24	0.68,7.41	0.185
COPD	0.97	0.24,3.87	0.969			
Cerebrovascular disease	0.39	0.13,1.21	0.104			
Obesity (BMI ≥ 30 kg/m2)	12.03	3.63,39.91	< 0.001			
Chronic kidney disease	0.66	0.24,1.76	0.406			
Chronic liver disease	1.77	0.28,11.02	0.540			
Solid organ transplant	2.64	0.16,43.26	0.496			
COVID-19	11.18	4.75,26.33	< 0.001	3.72	1.16,11.91	0.027
Previous hospitalization	0.72	0.34,1.50	0.379			
Previous antibiotic treatment	1.02	0.48,2.14	0.966			
Previous surgery	0.35	0.14,0.87	0.025			
ICU vs. Ward	19.11	7.57,48.27	< 0.001	8.83	2.94,26.51	< 0.001
Mechanical ventilation	15.35	6.34,37.18	< 0.001			
CVC	35.82	12.27,104.60	< 0.001			
Sites of colonization	1.14	1.02,1.28	0.022	1.01	0.86,1.17	0.948
Multisite of colonization	2.95	1.28,6.81	0.011			

*Abbreviations* BMI, body mass index; CI, confidence interval; CCI, Charlson Comorbidity Index; COPD, Chronic Obstructive Pulmonary Disease; COVID-19. Coronavirus disease; CVC, Central Venous Catheter; HIV, Human Immunodeficiency Virus; ICU, Intensive Care Unit, IQR, interquartile Range

AUROC of the model: 0.890 (95% CI 0.826–0.955). Model’s internal calibration evaluated with calibration belt test, *p* = 0.621

In the multivariable logistic regression analysis, cardiovascular disease (OR 5.83, 95% CI 1.12–30.43, *p* = 0.037), COVID-19 (OR 3.72, 95% CI 1.16–11.91, *p* = 0.027) and ICU admission (OR 8.83, 95% CI 2.94–26.51, *p* < 0.001) were all independent risk factors for the development of CRAB infection after CRAB colonization (Table 2).

## Discussion

In this study we observed that the burden of colonization and infection caused by CRAB changed significantly over the study period after the COVID-19 pandemic throughout the whole hospital and that cardiovascular disease, COVID-19 and ICU admission were significant risk factors associated with progression from CRAB colonization to CRAB infection.

During the Severe Acute Respiratory Syndrome Coronavirus 2 (SARS-CoV-2*)* pandemic, there were several nosocomial outbreaks caused by CRAB worldwide, with a significant impact on patients outcome [3, 19, 20]. In our study we observed a significant increase in colonization and infection rates both in general wards and ICU setting after the start of COVID-19 pandemic. The substantial representation of COVID-19 patients, constituting 34.6% of our cohort, underlines the pandemic effect on healthcare systems and the dynamic nature of MDR infection and colonization rates [19, 21]. Moreover, the high CRAB colonization rates observed in the general wards, suggest the need for implementing and reinforcing screening policies outside the ICU as well [22].

Infections due to MDR microorganisms may originate from a prior asymptomatic colonization status but the magnitude of this risk is poorly understood and needs better knowledge across different host types and pathogens [5–7, 23]. The risk of an MDR infection in patients colonized with CRAB was around 25% in our cohort. Although it is difficult to compare heterogeneous studies with variable populations, microorganisms, and surveillance methods, the risk of an MDR infection in patients colonized with MDR bacteria has been reported to be around 14% (range 8–44%), being between 11.6% and 44% for CRAB colonized patients [5–7, 24].

We found that cardiovascular disease was associated with a higher risk of infections in CRAB colonized patients. Cardiovascular disease is often part of a systemic inflammatory syndrome, highlighting the importance of a wider health definition including also the immunomodulatory effect of non-infectious disease conditions [25]. Our study confirms the negative effect of COVID-19 on predisposing colonized patients to the development of CRAB infections. Several pathophysiological factors could explain these findings, including SARS-CoV-2-mediated local and systemic immune paralysis, exposure to immunomodulatory agents and translocation phenomena related to local and systemic virus damage [26–28]. Moreover, we found that ICU setting significantly impacted the infection onset in colonized patients probably because of the use of invasive procedures (mechanical ventilation and CVC), which were not introduced in the multivariable analysis to avoid collinearities keeping the model more stable and reliable [6, 29, 30]. Altogether these predictors could be useful in prompting clinicians to start the most appropriate empirical therapy early in a case-by-case assessment. In contrast with former works in this line, the CCI and age resulted in protective factors for the development of clinical infections at univariate analysis [6, 29, 30]. This was likely due to the specific characteristics of our ICU population in the pandemic setting in Lombardia region, where ICU admission was based on best prognostic factors in survival to optimize resource utilization.

Overall hospital mortality was 38.9%, being 32.7 and 55% in colonized only and infected patients, respectively. These results underline that CRAB isolation might represent a marker of poor prognosis, regardless of clinical presentation, and highlight the frailty of the patient population carrying this pathogen [31]. Mortality of CRAB infected population was high, in keeping with the mortality rate reported in clinical studies ranging from 40 to 70%, depending on patients’ conditions, clinical severity, bacterial characteristics, and type of infection [6–9, 32, 33]. High mortality might be partly due to the very low rates of appropriate empirical antimicrobial therapy in our cohort since inactive empirical antibiotic therapy is a well-known risk factor for poor outcomes [34].

Our study has several limitations. Firstly, it is a single-center retrospective observational study and need for further prospective multicenter studies to validate our findings. Secondly, diagnosis of CRAB respiratory infections in patients with COVID-19 may be overestimated but our study reflects real-life challenges in distinguishing between colonization and infection. Thirdly, systematic surveillance screening is performed only in ICU ward in our hospital and colonization rates might be underestimated in non-ICU wards and might introduce an initial sample selection bias. Fourthly, the high proportion of COVID-19 patients poses challenges in terms of comparing our findings with prior research and generalizing our results to healthcare settings unaffected by SARS-CoV-2 infection. Fifthly, CRAB clonality was not confirmed but rather assumed if the CRAB isolated from surveillance culture had the same antibiotic resistance profile as the infecting CRAB, which reflects real-life practice.

In conclusion, our study provides a comprehensive analysis of CRAB colonization and infection in the whole nosocomial setting. We found an evolving epidemiology of CRAB infections and colonization throughout the COVID-19 pandemic highlighting the role of infection control measures and MDR monitorization in the hospital setting. CRAB colonized patients with cardiovascular disease, COVID-19 and admitted to ICU setting are a high-risk group for CRAB infections with the need of close monitoring. CRAB colonization and infection are associated with a high mortality rate. Future research should be prioritized to expand new stewardship programs and to implement targeted therapeutic interventions in CRAB colonized and infected patients.

## Electronic supplementary material

Below is the link to the electronic supplementary material.

Supplementary Material 1

## Data Availability

No datasets were generated or analysed during the current study.

## Abbreviations

AMR: Antimicrobial resistance
BMI: Body mass index
COVID-19: Coronavirus Disease 2019
CRAB: Carbapenem-resistant *Acinetobacter baumannii*
ICU: Intensive care unit
IQR: Interquartile range
MDR: Multidrug-resistant
SARS-CoV-2: Severe Acute Respiratory Syndrome Coronavirus 2
SD: Standard deviation
